# Multiomics reveals fatty acid metabolism and immune remodeling in retinal artery occlusion

**DOI:** 10.1016/j.isci.2026.116445

**Published:** 2026-06-17

**Authors:** Jiaqing Feng, Duan Chen, Chuansen Wang, Runlang Zhu, Longfei Chen, Liang Hu, Ting Chen, Ying Li, Xuan Xiao

**Affiliations:** 1 Department of Ophthalmology, Renmin Hospital of Wuhan University, Wuhan 430060, Hubei, China; 2 Department of Clinical Laboratory, Institute of Translational Medicine, Renmin Hospital of Wuhan University, Wuhan 430060, Hubei, China

**Keywords:** biological sciences

## Abstract

Retinal artery occlusion (RAO), an acute ocular ischemic stroke, is critically linked to cardiovascular and metabolic dysfunction. To elucidate the interplay between lipid metabolism and immune regulation in RAO, we performed an integrated analysis using serum metabolomics, PBMC transcriptomics, and machine learning in 66 patients with RAO and 66 cataract controls. We identified systemic disruptions in fatty acid metabolism, notably elevated levels of long-chain fatty acids (C22:5*n*-3, C22:2*n*-6, C22:1*n*-9), as distinguishing features of RAO. Upon stratifying patients by lipid status, C20:2*n*-6 emerged as the top biomarker distinguishing dyslipidemic RAO from non-dyslipidemic cases. Multiomics analysis correlated this accumulation with specific immune pathways, particularly Treg modulation. In Treg-like MT-2 cells, C20:2*n*-6 promoted Treg proliferation and induced the secretion of the anti-inflammatory cytokine IL-10, independent of TGF-β. These findings highlight C20:2*n*-6 as a candidate biomarker and implicate the C20:2n-6–Treg–IL-10 axis as a therapeutic target for restoring immune homeostasis in dyslipidemia-associated RAO.

## Highlights

• Multiomics reveals dysregulated fatty acid and immune profiles in RAO
• Machine learning identifies C20:2n-6 as a specific signature of dyslipidemic RAO
• C20:2n-6 specifically promotes Treg proliferation and IL-10 secretion *in vitro*
• The C20:2n-6–Treg–IL-10 axis represents a potential immunometabolic therapeutic target

## Graphical abstract

**Figure:**
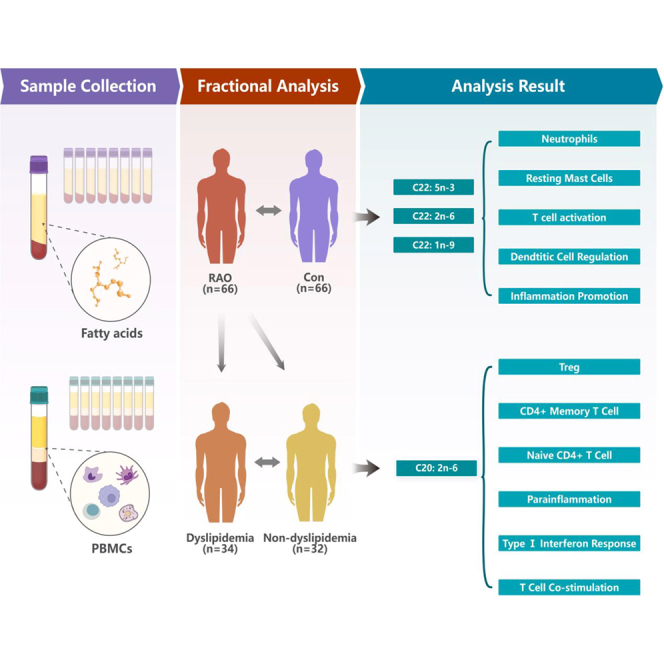

## Introduction

Retinal artery occlusion (RAO) is an acute retinal ischemic event that leads to sudden vision loss. Although the annual incidence of RAO is estimated to be 1.8–2.5 cases per 100,000 individuals, only limited therapeutic options are currently available.^1^^,^^2^ The clinical importance of RAO extends beyond ocular morbidity, as evidenced by its recognition by the American Heart Association as a marker of cerebrovascular and cardiovascular events.^1^ In a previous study, we demonstrated that 23.4% of patients with RAO are at a risk of major adverse cardio-cerebrovascular events within three years,^3^ highlighting the clinical significance of understanding the underlying mechanisms. The pathological similarities between RAO and systemic cardio-cerebrovascular diseases, particularly in their shared progression of atherosclerosis, suggest common pathogenic pathways.^3^ Similar to other arterial occlusive disorders, RAO is strongly associated with traditional vascular risk factors, including hypertension, diabetes mellitus, dyslipidemia, elevated inflammatory biomarkers, and hemorheological disturbances.^4^^,^^5^ Among these, dyslipidemia and associated disturbances in fatty acid metabolism have emerged as important contributors to the pathogenesis of RAO via their effects on endothelial dysfunction, thrombogenesis, and systemic inflammation.^6^^,^^7^

Fatty acid metabolism involves complex processes, including synthesis, oxidation, and transformation, with dysregulation potentially leading to significant alterations in lipid profiles.^2^^,^^8^ Growing evidence suggests that fatty acids are not only metabolic intermediates but also serve as signaling molecules that influence immune responses and inflammatory pathways in vascular occlusion diseases.^9^^,^^10^^,^^11^ Differential associations between specific fatty acids and vascular outcomes have been reported. Borges et al. reported an association between linoleic acid and a decreased risk of stroke, but found that monounsaturated fatty acids were linked to an increased risk of coronary heart disease.^12^ In cerebral ischemia models, short-chain fatty acids (SCFAs) interact with both the peripheral and central nervous systems. They can exert direct or indirect effects by activating the immune and autonomic nervous systems, stimulating microglial activation, and modulating the integrity of the blood-brain barrier (BBB).^13^^,^^14^ Notably, medium- and long-chain fatty acids (MLCFAs) exhibit dual roles in ischemic pathologies, with omega-3 polyunsaturated fatty acids demonstrating anti-inflammatory properties and saturated MLCFAs potentially exacerbating oxidative stress via mitochondrial dysfunction.^14^^,^^15^^,^^16^ Although the involvement of fatty acid metabolism in vascular occlusive disorders has been well documented, the specific alterations in the fatty acid metabolome and their functional consequences in RAO remain unexplored.

Beyond abnormal fatty acid metabolic pathways in vascular occlusive diseases, emerging evidence indicates that peripheral immune cells and their components play pivotal roles in the pathogenesis of ischemic stroke, demonstrating dichotomous functions in both exacerbating injury and promoting repair processes.^17^^,^^18^^,^^19^^,^^20^ In previous studies, we identified alterations in immune cell subsets in the peripheral blood of patients with RAO, suggesting a similar peripheral immunological paradigm in the pathogenesis of RAO.^21^ Recent metabolomic studies on systemic vascular diseases have revealed disease-specific fatty acid signatures associated with immune regulation^22^^,^^23^^,^^24^; however, such investigations are lacking for RAO.

To address these gaps, in this study, we integrated targeted serum fatty acid metabolomics with peripheral blood mononuclear cell (PBMC) transcriptomics for patients with RAO. We employed multiple machine-learning approaches to robustly identify diagnostic metabolic biomarkers specific to RAO and its dyslipidemic subtype. Moving beyond correlational analysis, we experimentally validated the functional impact of the identified key fatty acids on immune cell plasticity using an *in vitro* regulatory T cell model. By elucidating the mechanistic link between specific fatty acid signatures and peripheral immune regulation, we aimed to establish the C20:2n-6–Treg–IL-10 axis as a potential therapeutic target and provide new insights into the immunometabolic pathogenesis of RAO.

## Results

### Clinical characteristics of the study sample

Table 1 shows the anthropometric, clinical, and biochemical characteristics of the study participants. A total of 132 participants—66 patients with RAO and 66 cataract control individuals—were enrolled. The patients with RAO and control individuals were well matched in terms of age, sex, hypertension, and diabetes mellitus (*p* > 0.05 for each group), indicating comparable baseline characteristics.

**Table 1:** Baseline demographic characteristics between the retinal artery occlusion and control groups

Variable	Retinal artery occlusion (*n* = 66)	Control (*n* = 66)	x^2^/Z/t	*p*
Age (years)	66.50 (56.75, 70.00)	66.50 (57.00, 71.00)	0.371	0.710
Sex (Male)	37	31	1.092	0.296
Hypertension	33	25	1.968	0.161
Diabetes	8	8	<0.001	1.000
Triglyceride (mmol/L)	1.62 (1.09, 1.96)	1.12 (0.89, 1.61)	3.088	**0.002**
Total cholesterol (mmol/L)	4.76 (4.07, 5.43)	4.64 (3.96, 5.23)	0.712	0.476
High-density lipoprotein cholesterol (mmol/L)	1.07 (0.90, 1.26)	1.16 (0.96, 1.38)	1.771	0.077
Low-density lipoprotein cholesterol (mmol/L)	2.94 (2.47, 3.40)	2.83 (2.27, 3.48)	0.721	0.471
Small dense LDL cholesterol (mmol/L)	0.88 (0.61, 1.17)	0.74 (0.57, 0.93)	1.775	0.076
Total/HDL cholesterol	4.41 (3.78, 5.14)	3.99 (3.28, 4.54)	2.389	**0.017**
Atherogenic index	3.41 (2.78, 4.15)	2.99 (2.28, 3.54)	2.396	**0.017**
ApoA1 (g/L)	1.29 (1.17, 1.39)	1.30 (1.19, 1.50)	1.161	0.246
ApoB (g/L)	1.45 ± 0.34	1.57 ± 0.44	1.839	0.068
ApoA1/B	1.38 (1.22, 1.65)	1.46 (1.27, 1.85)	1.627	0.104
Lp(a) (g/L)	175.35 (87.25, 359.08)	185.10 (83.16, 464.60)	0.560	0.576

Data in bold represent statistically significant differences (*p* < 0.05).

We compared the comprehensive lipid profiles between patients with RAO and control subjects with cataracts. The following parameters were measured: TG, TC, LDL-C, HDL-C, sdLDL, total/HDL cholesterol ratio, atherogenic index, ApoA1, ApoB, and lipoprotein(a) [Lp(a)]. According to the Chinese Guidelines for the Management of Dyslipidemia in Adults,^25^ the normal reference ranges for each lipid parameter are as follows: TG < 1.69 mmol/L, TC < 5.2 mmol/L, HDL-C ≥ 1.04 mmol/L, LDL-C < 3.4 mmol/L, sdLDL-C < 1.0 mmol/L, TC/HDL-C ratio <4.5, atherogenic index <3.3, ApoA1 1.20–1.60 g/L, ApoB 0.80–1.20 g/L, and Lp(a) < 300 mg/L.

Statistical analysis revealed that among the lipid parameters assessed, TG levels (*p* = 0.002) and atherogenic index (*p* = 0.017) were significantly higher in the RAO group than in controls, despite TG levels remaining within the upper limit of the normal reference range. Total/HDL cholesterol ratio was significantly elevated (*p* = 0.017), indicating a relative reduction in HDL levels within the RAO lipid profile. These findings suggest that higher TG levels and relatively lower HDL levels are clinically significant features in the RAO population, with the elevated atherogenic index potentially indicating a higher risk for RAO.

### Patients with RAO exhibit corresponding changes in fatty acid metabolism

**Figure 1:**
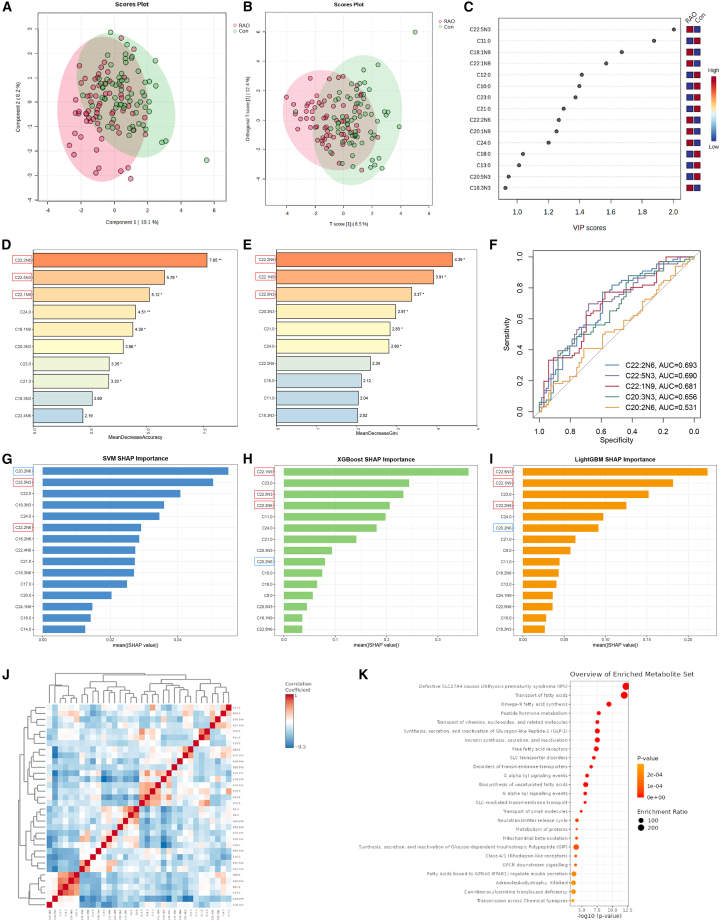
Systematic identification and characterization of serum fatty acid signatures in retinal artery occlusion (A and B) Score plots of sPLS-DA and OPLS-DA models. Red indicates the RAO group, whereas green indicates the Con group. (C) VIP scores derived from the OPLS-DA model, with red/blue squares indicating high/low relative abundance in the RAO vs. Con groups. (D–I) Predictive performance and feature importance analysis based on machine learning models. Feature importance rankings from random forest analysis based on MeanDecreaseAccuracy (D) and MeanDecreaseGini (E). (F) ROC curves of selected fatty acid indicators with AUC values >0.65. SHAP importance plots for SVM (G), XGBoost (H), and LightGBM (I), where bar length represents the mean absolute SHAP value. Boxed areas highlight key model-specific biomarkers. (J) Correlation heatmap illustrating the inter-relationships among identified fatty acids. (K) Pathway enrichment analysis via RaMP-DB based on differential metabolites. ∗*p* < 0.05 and ∗∗*p* < 0.01 by response-permutation test of random forest variable importance (rfPermute, nrep = 1000) for MeanDecreaseAccuracy (D) and MeanDecreaseGini (E).

To investigate the alterations in fatty acid metabolism in patients with RAO, we performed multivariate statistical analyses, including sPLS-DA and OPLS-DA, for evaluating the discriminatory potential of the serum lipidomic profile. Both models showed a clear trend of separation between patients with RAO and control individuals (Figures 1A and 1B), suggesting identifiable shifts in the serum fatty acid landscape. Thirteen fatty acids were further identified as metabolic differentials that contribute most to this group variance (OPLS-DA model, VIP >1, *p* < 0.05) (Figure 1C). Notably, fatty acids, such as C22:5*n*-3, C18:1*n*-9, and C22:1*n*-9, emerged as the leading contributors to the OPLS-DA model, exhibiting the highest VIP scores among all identified differential metabolites. This prioritized ranking underscores their potential significance as candidate biomarkers for RAO.

To further identify key fatty acid metabolites responsible for group discrimination and to quantify their predictive robustness, we applied machine-learning models, including Random Forest (RF), SVM, XGBoost, and LightGBM, based on the 13 fatty acid indicators identified. The RF analysis was applied to evaluate the importance of these indicators in classifying patients with RAO; the feature importance rankings derived from mean decrease accuracy (Figure 1D) and mean decrease in Gini (Figure 1E) consistently highlighted C22:2*n*-6, C22:1*n*-9, and C22:5*n*-3 as the most influential indicators. The predictive performance was evaluated using ROC curves, where C22:2*n*-6, C22:5*n*-3, C22:1*n*-9, and C20:3*n*-3 demonstrated strong discriminatory power with AUC values exceeding 0.65 (Figure 1F). Furthermore, SHAP importance analysis across SVM, XGBoost, and LightGBM models identified C22:2*n*-6 and C22:5*n*-3 as top-ranked biomarkers (Figures 1G–1I). Notably, the SVM model uniquely identified C20:2*n*-6 as the top-ranking predictive biomarker.

Following the machine-learning validation, correlation analysis was conducted to examine the relationships among these identified serum fatty acids, revealing complex synergistic or antagonistic associations (Figure 1J). Finally, to identify enriched metabolic pathways, fatty acids with VIP >1 were analyzed via RaMP-DB. The results revealed several significantly enriched pathways, including omega-9 fatty acid synthesis, transport of fatty acids, and synthesis of glucagon-like peptides (Figure 1K).

### Fatty acid metabolomics and PBMC transcriptomics reveal key immune-related pathways in patients with RAO

Building on the established link between fatty acid metabolism and immune regulation, and our previous observations of altered immune cell infiltration in RAO,^21^ we examined the hematological and immunological profiles of the RAO and control groups. Although the mean values for both cohorts remained within established physiological reference ranges, patients with RAO exhibited a statistically significant shift in their immune baseline. In particular, we observed a relative elevation in the percentage (*p* = 0.001) and absolute count (*p* = 0.004) of neutrophils, coupled with a reduction in lymphocyte percentage (*p* = 0.001) and eosinophil counts (*p* = 0.046) (Table 2). These findings suggest an identifiable and consistent systemic immune recalibration associated with RAO, rather than a transient physiological fluctuation.

**Table 2:** Characteristics of immune cell-related indicators between the retinal artery occlusion and control groups

Variable	Retinal artery occlusion (*n* = 66)	Control (*n* = 66)	x^2^/Z/t	*p*
White blood count (×10^9^/L)	6.19 ± 1.67	5.71 ± 1.40	1.780	0.077
Neutrophil (%)	59.00 (50.93, 65.00)	52.65 (48.20, 57.95)	3.332	**0.001**
Neutrophil count (×10^9^/L)	3.61 ± 1.22	2.90 (2.43, 3.73)	2.915	**0.004**
Monocyte (%)	7.50 (6.70, 8.60)	7.75 (6.98, 8.63)	1.068	0.286
Monocyte count (×10^9^/L)	0.48 (0.36, 0.58)	0.43 (0.37, 0.53)	0.993	0.321
Lymphocyte (%)	29.10 (25.55, 35.43)	35.12 ± 7.55	3.254	**0.001**
Lymphocyte count (×10^9^/L)	1.72 (1.48, 2.34)	1.98 ± 0.58	0.872	0.383
Eosinophil (%)	1.70 (1.10, 2.83)	2.20 (1.40, 3.23)	−1.250	0.214
Eosinophil count (×10^9^/L)	0.11 (0.07, 0.17)	0.13 (0.09, 0.23)	−2.036	**0.046**
Basophil (%)	0.45 (0.30, 0.60)	0.50 (0.30, 0.70)	−1.673	0.097
Basophil count (×10^9^/L)	0.03 (0.02, 0.04)	0.03 (0.02, 0.04)	−0.620	0.537

Data in bold represent statistically significant differences (*p* < 0.05).

**Figure 2:**
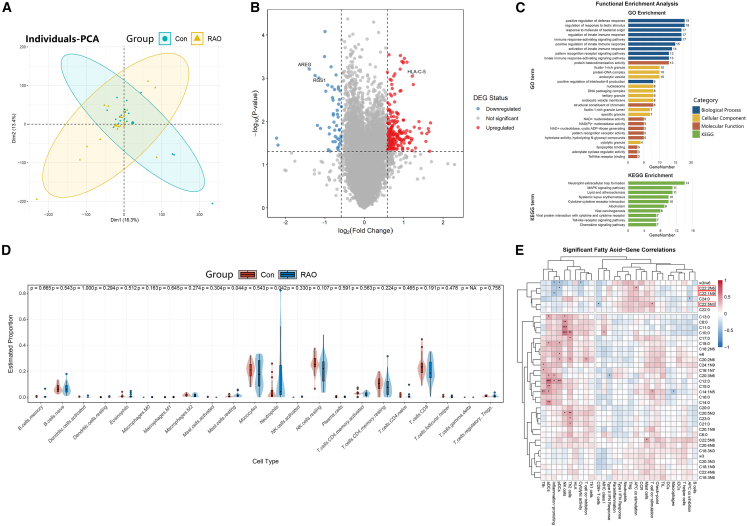
Integrated fatty acid metabolomics and peripheral blood mononuclear cell transcriptomics (A) PCA plot of venous blood samples. Yellow dots represent RAO venous blood samples, while blue dots represent control venous blood samples. (B) Volcano plot displaying DEGs between patients with RAO and controls. (C) GO and KEGG enrichment analysis of DEGs. (D) The proportion of immune cell populations in RAO vs. control group. Red parts represent cataract controls. Blue parts represent patients with RAO. (E) Heatmap of correlations between immune signatures and fatty acids. ∗*p* < 0.05, ∗∗*p* < 0.01, and ∗∗∗*p* < 0.001 by limma moderated *t* test (B), by Mann–Whitney U test (D), or by Spearman’s rank correlation test (E).

To systematically investigate peripheral immune mechanisms in RAO, we performed RNA-seq on PBMCs from 19 patients with RAO and 18 cataract controls. PCA revealed a partial separation between patients with RAO and controls in the transcriptomic profiles (Figure 2A). We identified 258 differentially expressed genes (DEGs), including upregulated HLA-C-5 and FOS, along with downregulated AREG and RGS1 (Figure 2B). Functional enrichment analysis revealed concurrent activation of innate immune pathways and alterations in NAD^+^ nucleosidase activity-related metabolic pathways (Figure 2C). Analysis of immune cell infiltration demonstrated a significant increase in resting mast cells and neutrophils in the RAO group (*p* < 0.05) compared with the respective numbers in controls (Figure 2D). Both pathway and immune infiltration analyses consistently indicated a distinct proinflammatory pattern in RAO. Furthermore, to elucidate the interplay between fatty acid metabolism and immune regulation, we performed an integrated multiomics analysis combining PBMC transcriptomic profiling with targeted fatty acid metabolomics. Pearson’s correlation analysis revealed a significant association (|r| = 0.75, *p* ≤ 0.05) between specific fatty acids and immune-related gene signatures (Figure 2E). Notably, among the previously identified fatty acid biomarkers, we observed distinct immunomodulatory correlations. C22:2*n*-6 exhibited a negative correlation with plasmacytoid dendritic cells (pDCs) but a positive association with APC costimulation, suggesting a dual role in dendritic cell regulation. C22:1*n*-9 was significantly linked to proinflammatory responses, with potential implications in the promotion of inflammation. C22:5*n*-3 showed a negative correlation with CD8^+^ T cell infiltration but a positive correlation with T cell costimulation, indicating a possible immunoregulatory function in T cell activation. C20:2*n*-6 displayed positive correlation with pDCs, Th2 cells, and T cell coinhibition, implying its involvement in Th2-polarized immune suppression.

### Identification of C20:2n-6 as a core metabolic signature in dyslipidemia in patients with RAO

Dyslipidemia may be a significant risk factor for cardiovascular and cerebrovascular obstructive diseases.^17^^,^^18^ To investigate whether dyslipidemia influences fatty acid metabolism in patients with RAO, we stratified them into dyslipidemia (*n* = 34) and non-dyslipidemia (*n* = 32) groups based on lipid profile abnormalities (Table 3).

**Table 3:** Blood lipids and related indices in patients with retinal artery occlusion exhibiting dyslipidemia

Variable	Retinal artery occlusion (*n* = 66)		x^2^/Z/t	*p*
	Dyslipidemia (*n* = 34)	Non-dyslipidemia (*n* = 32)		
Age (years)	61.91 ± 10.01	64.16 ± 10.22	−0.900	0.371
Sex (Male)	19	13	6.008	**0.014**
Hypertension	18	15	0.243	0.622
Diabetes	3	5	0.716	0.397
Triglyceride (mmol/L)	2.06 ± 0.99	1.37 ± 0.41	3.730	**0.001**
Total cholesterol (mmol/L)	4.65 ± 1.05	4.93 ± 0.64	−1.327	0.190
High-density lipoprotein cholesterol (mmol/L)	0.93 ± 0.15	1.27 ± 0.21	−7.727	**0.001**
Low-density lipoprotein cholesterol (mmol/L)	2.92 ± 0.84	2.99 ± 0.66	−0.365	0.716
Small dense LDL cholesterol (mmol/L)	1.04 ± 0.40	0.78 ± 0.30	3.022	**0.004**
Total/HDL cholesterol	5.03 ± 0.94	3.94 ± 0.70	5.290	**0.001**
Atherogenic index	4.03 ± 0.94	2.94 ± 0.70	5.291	**0.001**
ApoA1 (g/L)	1.21 ± 0.13	1.40 ± 0.14	−5.943	**0.001**
ApoB (g/L)	0.95 ± 0.22	0.92 ± 0.15	0.465	0.643
ApoA1/B	1.33 ± 0.29	1.57 ± 0.35	−3.014	**0.004**
Lp(a) (g/L)	198.38 ± 184.82	322.84 ± 267.96	−2.184	**0.033**

Data in bold represent statistically significant differences (*p* < 0.05).

**Figure 3:**
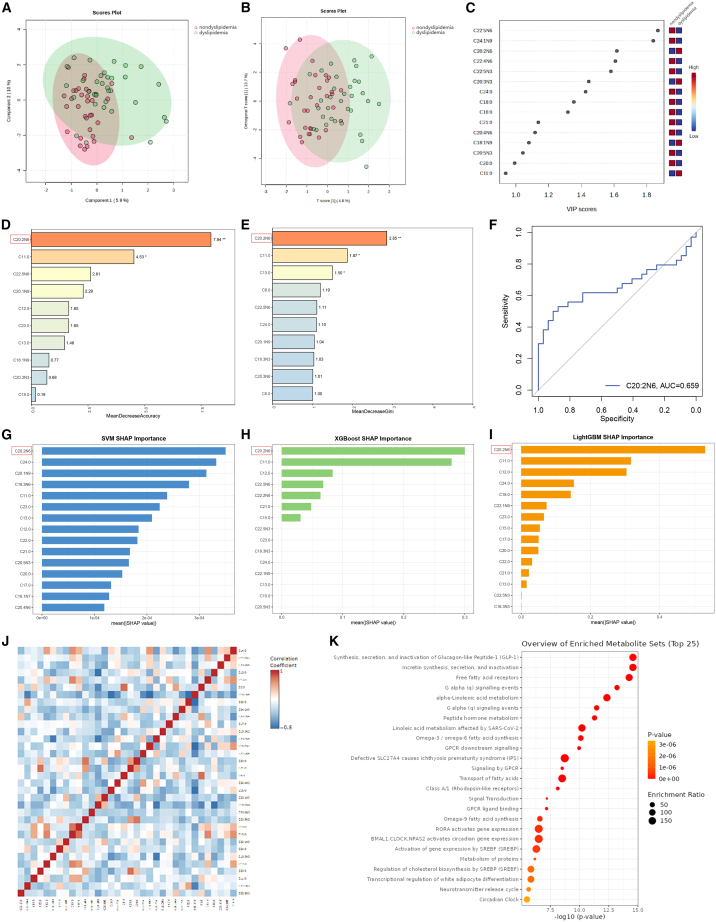
Identification and characterization of fatty acid signatures in patients with retinal artery occlusion exhibiting dyslipidemia (A and B) Score plots of sPLS-DA and OPLS-DA models. Red indicates non-dyslipidemic patients with RAO, and green indicates patients with dyslipidemic RAO. (C) VIP scores derived from the OPLS-DA model, with red/blue squares indicating high/low relative abundance in the dyslipidemia vs. non-dyslipidemia groups. (D–I) Predictive performance and feature importance analysis based on machine learning models. Feature importance rankings from random forest analysis based on MeanDecreaseAccuracy (D) and MeanDecreaseGini (E). (F) ROC curve of C20:2*n*-6. SHAP importance plots for SVM (G), XGBoost (H), and LightGBM (I), where bar length represents the mean absolute SHAP value. (J) Correlation heatmap illustrating the inter-relationships among identified fatty acids. (K) Pathway enrichment analysis via RaMP-DB based on differential metabolites. ∗*p* < 0.05 and ∗∗*p* < 0.01 by response-permutation test of random forest variable importance (rfPermute, nrep = 1000) for MeanDecreaseAccuracy (D) and MeanDecreaseGini (E).

To further delineate the metabolic alterations specific to dyslipidemia, we performed a targeted analysis within the RAO cohort. Both sPLS-DA and OPLS-DA models revealed distinct shifts in fatty acid composition between the dyslipidemia and non-dyslipidemia RAO groups (Figures 3A and 3B). In particular, VIP analysis identified C20:2*n*-6, a key member of the omega-6 fatty acid family, as a distinguishing metabolite significantly elevated in the dyslipidemia RAO group (Figure 3C).

To validate the importance of these metabolic shifts, we employed a suite of integrated machine-learning algorithms. Across multiple computational frameworks, including RF analysis based on mean decrease accuracy and mean decrease in Gini (Figures 3D and 3E), ROC curve evaluation (Figure 3F), and SHAP value assessments applied to SVM, XGBoost, and LightGBM models (Figures 3G–3I), C20:2*n*-6 was consistently identified as the most influential feature. The results from these independent models converged on C20:2*n*-6 as the leading diagnostic marker, highlighting the stability of this metabolic signature across different algorithmic approaches.

Furthermore, correlation analysis revealed the interconnected metabolic relationships associated with these identified signatures (Figure 3J). This was complemented by enrichment analysis, which highlighted systematic dysregulation across multiple pathways, including the omega-3 and omega-6 fatty acid synthesis and metabolism (Figure 3K). Collectively, the consistent prioritization of C20:2*n*-6 across various modeling strategies and its integration into fatty acid metabolic networks point to its role in the systemic lipid remodeling characteristic of patients with RAO exhibiting dyslipidemia.

### Alterations in fatty acid-immune regulatory networks in patients with RAO exhibiting dyslipidemia

To elucidate the impact of dyslipidemia on fatty acid metabolism and immune regulation in RAO progression, we performed comparative multiomics analysis of 66 patients with RAO stratified by lipid status (dyslipidemia, *n* = 34; non-dyslipidemia, *n* = 32). Initial immunophenotyping revealed comparable peripheral immune cell distributions between groups (Table 4), suggesting that disturbances in lipid metabolism may preferentially affect functional states rather than cellular composition.

**Table 4:** Characteristics of immune cell-related indicators in dyslipidemia and non-dyslipidemia groups of patients with retinal artery occlusion

Variable	Retinal artery occlusion (*n* = 66)		x^2^/Z/t	*p*
	Dyslipidemia (*n* = 34)	Non-dyslipidemia (*n* = 32)		
White blood count (×10^9^/L)	6.17 ± 1.70	6.21 ± 1.57	0.086	0.932
Neutrophil (%)	58.50 (50.78, 64.53)	59.45 (51.25, 65.65)	0.276	0.783
Neutrophil count (×10^9^/L)	3.61 ± 1.18	3.68 ± 1.27	0.228	0.821
Monocyte (%)	7.85 (6.88, 8.93)	7.15 (6.40, 8.30)	1.721	0.085
Monocyte count (×10^9^/L)	0.50 ± 0.15	0.48 (0.36, 0.55)	0.758	0.449
Lymphocyte (%)	28.85 (25.85, 35.98)	29.35 (25.18, 35.20)	0.263	0.793
Lymphocyte count (×10^9^/L)	1.68 (1.45, 2.36)	1.94 ± 0.60	0.809	0.419
Eosinophil (%)	1.75 (1.28, 2.65)	1.60 (1.00, 3.05)	0.494	0.621
Eosinophil count (×10^9^/L)	0.11 (0.07, 0.17)	0.10 (0.07, 0.18)	0.289	0.772
Basophil (%)	0.45 (0.40, 0.60)	0.44 ± 0.20	1.072	0.284
Basophil count (×10^9^/L)	0.02 (0.02, 0.43)	0.03 (0.02, 0.04)	0.758	0.448

**Figure 4:**
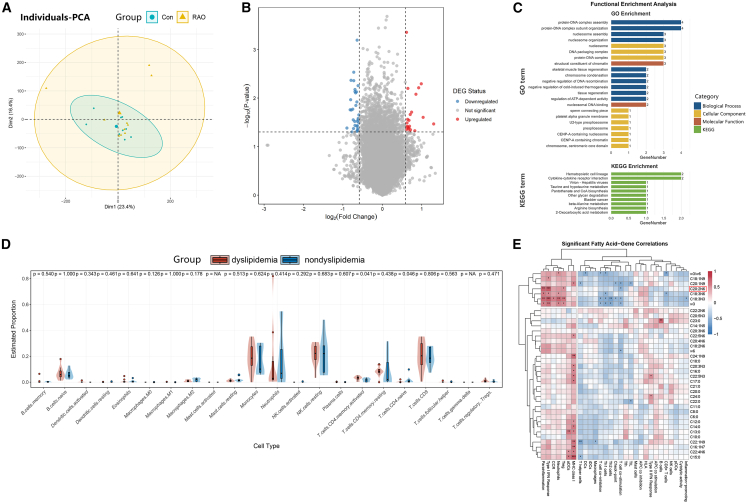
Integrated analysis of fatty acid metabolomics and peripheral blood mononuclear cell transcriptomics (A) PCA plot of patients with RAO stratified by lipid status. Yellow dots represent patients with dyslipidemia RAO, while blue dots represent patients with non-dyslipidemia RAO. (B) Volcano plot displays DEGs between patients with dyslipidemia and non-dyslipidemia. (C) GO and KEGG enrichment analysis of DEGs. (D) The proportion of immune cell populations in patients with dyslipidemic and non-dyslipidemic RAO. Red parts represent patients with dyslipidemic. Blue parts represent patients with non-dyslipidemic. (E) Heatmap of correlations between immune signatures and fatty acids. ∗*p* < 0.05, ∗∗*p* < 0.01, and ∗∗∗*p* < 0.001 by limma moderated *t* test (B), by Mann–Whitney U test (D), or by Spearman’s rank correlation test (E).

To elucidate how lipid metabolic dysregulation, particularly the accumulation of C20:2*n*-6, affects immune cell functional states in RAO pathogenesis, we conducted stratified transcriptomic profiling of PBMCs from 19 matched patients. Our analysis revealed significant lipid-status-dependent reprogramming. PCA and DEG analysis (Figures 4A and 4B) identified distinct clustering and 53 DEGs. Pathway enrichment revealed significant alterations in genes encoding structural constituents of chromatin and tissue repair processes (Figure 4C). Immune deconvolution revealed lipid-specific T cell modulation, characterized by enhanced CD4^+^ memory T cell activation (*p* = 0.041) and decreased naive CD4^+^ T cell infiltration (*p* = 0.046) (Figure 4D). Building on our previous finding that C20:2*n*-6 strongly associates with dyslipidemia in RAO, we specifically investigated its immunomodulatory effects via multiomics analysis. The results indicated that C20:2*n*-6 exhibits a strong positive correlation with Treg, parainflammation, and type I interferon response pathways, but a negative correlation with T cell costimulation, indicative of its central role in reshaping immune responses under dyslipidemic conditions (Figure 4E).

### C20:2n-6 promotes proliferation and IL-10 secretion in MT-2 cells

**Figure 5:**
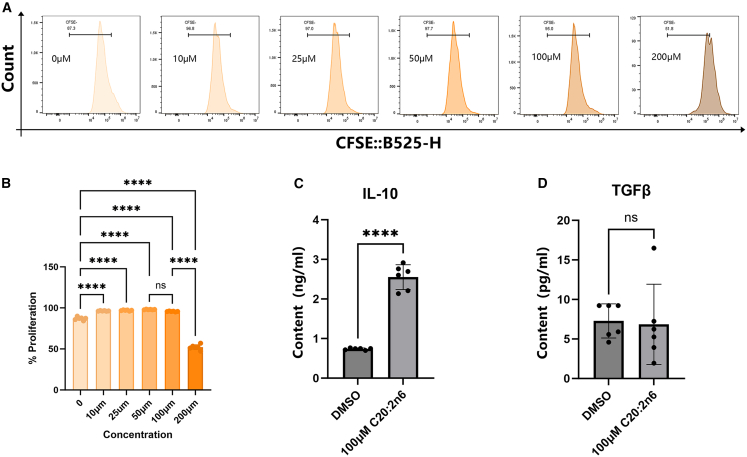
C20:2*n*-6 modulates the proliferation of MT-2 cells and cytokine secretion (A) Representative flow cytometry histograms of CFSE-labeled MT-2 cells treated with indicated concentrations of C20:2*n*-6 (0, 10, 25, 50, 100, and 200 μM). (B) Statistical analysis of MT-2 cell proliferation (%) across the concentration gradient. (C and D) ELISA quantification of IL-10 (C) and TGF-β (D) levels in the supernatant of MT-2 cells treated with 100 μM C20:2*n*-6 or DMSO control. Data are presented as mean ± SD (*n* = 6 biologically independent samples). ∗∗∗∗*p* < 0.0001, ns (not significant) by one-way ANOVA (B) or by Student’s *t* test (C and D).

To experimentally validate the potential immunomodulatory role of C20:2*n*-6, we utilized the MT-2 cell line, which constitutively expresses FoxP3 and CD25 and is widely recognized as a suitable *in vitro* model for studying human Treg function.^26^^,^^27^ We first evaluated the impact of a C20:2*n*-6 concentration gradient (0–200 μM) on cell proliferation. CFSE-based flow cytometry revealed that C20:2*n*-6 significantly stimulated MT-2 proliferation in a dose-dependent manner up to 100 μM (*p* < 0.01, Figures 5A and 5B). However, cell proliferation was markedly suppressed at a higher concentration of 200 μM compared with that in the 100 μM group (*p* < 0.0001, Figure 5B).

Based on these results, 100 μM C20:2*n*-6 was selected for cytokine secretion analysis. ELISA results showed that 100 μM C20:2*n*-6 intervention significantly increased the secretion of IL-10 (*p* < 0.0001, Figure 5C). Notably, C20:2*n*-6 treatment did not induce significant changes in TGF-β levels (Figure 5D), suggesting that IL-10 may serve as a specific downstream effector through which C20:2*n*-6 modulates the immune activity of MT-2 cells. These findings provide direct experimental evidence that C20:2*n*-6 enhances the expansion and specific anti-inflammatory secretory function of Treg-like cells.

## Discussion

In this study, we deciphered, for the first time, the dynamic link between metabolic dysregulation and immune imbalance during RAO progression by integrating peripheral serum fatty acid metabolomics, PBMC transcriptomics, and immune phenotyping.

### Systemic fatty acid dysregulation and metabolic-immune crosstalk

At the metabolomics level, patients with RAO exhibited significantly elevated levels of three long-chain fatty acids: C22:5*n*-3, C22:2*n*-6, and C22:1*n*-9. Notably, while the associated hematological parameters remained within the established physiological ranges, these consistent statistical shifts reflect a systemic immune “re-tuning” rather than overt pathology. This maintenance of homeostatic cell counts suggests that RAO-associated fatty acid dysregulation may primarily modulate the functional potency of immune cells rather than driving massive numerical fluctuations. Such qualitative reprogramming aligns with the results of our subsequent transcriptomic analysis, wherein predominant enrichment of genes involved in innate immunity and functional activation pathways was observed. These systemic deviations, though subclinical in magnitude, highlight an identifiable immune recalibration primed by metabolic cues during acute ischemic stress. Moreover, our correlation analysis showed potential regulatory effects of fatty acids on immune subpopulations, indicative of active modulation of the immune microenvironment by fatty acids via multiple functional pathways.

### Immunomodulatory roles of C22 fatty acids

Our further analysis shows that an elevation in the omega-3 polyunsaturated fatty acid C22:5*n*-3 likely reflects a compensatory response to acute ischemia and exhibits concentration-dependent dual effects. At low concentrations, C22:5*n*-3 protects against damage by inhibiting oxidative stress and preserving the integrity of the neurovascular unit.^13^ In contrast, at high concentrations, it competitively inhibits cyclooxygenase, thereby promoting the formation of the prothrombotic mediator TXA2.^12^ This paradox is consistent with clinical findings regarding mortality and myocardial infarction risks.^28^^,^^29^ We observed that the accumulation of C22:1*n*-9 suppressed fatty acid oxidation, which leads to lipid deposition and mitochondrial dysfunction, ultimately creating a locally hypoxic microenvironment.^24^^,^^30^ Additionally, the increase in the levels of C22:2*n*-6, an omega-6 polyunsaturated fatty acid, indicates hypoxia-driven reprogramming. This molecule extends from arachidonic acid via a FADS1-dependent pathway and may jointly exacerbate retinal ischemia by upregulating FADS1 through a positive feedback loop.^31^^,^^32^

### Specific role of C20:2n-6 in the dslipidemia sbgroup

In the dyslipidemia subgroup, we observed a disease-specific increase in the levels of C20:2*n*-6. Unlike the increase in general obesity-related free fatty acids, C20:2*n*-6 accumulates specifically in RAO with dyslipidemia,^33^^,^^34^ possibly as a consequence of enhanced release by adipose tissue combined with inhibited local FADS2 activity.^35^ Although previous mechanistic studies have demonstrated that macrophages can take up C20:2*n*-6 and metabolize it into proinflammatory and proangiogenic mediators,^36^ its role in modulating adaptive immune subsets in RAO remained largely unexplored.

To further substantiate our multiomics findings, we conducted functional assays using the MT-2 cell line, a widely recognized model for human Tregs.^26^^,^^27^ C20:2*n*-6 treatment significantly promoted the proliferation of MT-2 cells at an optimal concentration of 100 μM and specifically induced the secretion of the anti-inflammatory cytokine IL-10, whereas TGF-β levels remained unchanged (Figure 5). This selective induction suggests that C20:2*n*-6 may specifically target the IL-10 signaling axis, potentially via the activation of lipid-sensitive transcription factors such as PPARs.^37^ Our experimental findings are corroborative of the emerging evidence that omega-6 polyunsaturated fatty acids are critical regulators of the metabolic fitness of T cells and memory differentiation.^38^

In diabetic retinopathy models, Treg expansion protects against neuronal loss.^39^ However, in cerebral ischemia models, Tregs may worsen thromboinflammation via LFA-1/ICAM-1 interactions.^40^ Thus, the pro-Treg and anti-inflammatory effects of C20:2*n*-6 via IL-10 may be context-dependent, potentially serving as an endogenous metabolic checkpoint to resolve acute immune activation triggered by ischemia. Thus, further investigations using *in vivo* RAO models are warranted to validate the precise role of the C20:2n-6–Treg–IL-10 axis in the spatiotemporal progression of RAO.

### Synergistic and antagonistic interactions within metabolic networks

Evidence suggests that, in biological systems, fatty acids do not function in isolation but operate within an integrated metabolic-immune network. Our pathway enrichment results revealed significant alterations across omega-3, omega-6, and omega-9 synthesis pathways (Figures 1K and 3K). Beyond the enzymatic competition for FADS1/2 and ELOVL5—where an increase in omega-6 intermediates such as C20:2*n*-6 can competitively inhibit the flux toward protective omega-3 derivatives—these metabolites exhibit complex synergistic interactions. In particular, the co-elevation of very-long-chain fatty acids (VLCFAs), such as C22:5*n*-3 and C22:2*n*-6, may collectively alter the organization of lipid rafts, thereby modulating receptor clustering during T cell costimulation.^41^^,^^42^ Furthermore, the interaction between omega-9 (C22:1*n*-9) and polyunsaturated fatty acids suggests an additional layer of crosstalk; the accumulation of monounsaturated VLCFAs can interfere with the β-oxidation efficiency of other lipids, leading to a synergistic increase in mitochondrial stress.^24^^,^^30^ Such multifactorial interactions determine the integrated lipid landscape and the subsequent immune response in RAO.

In summary, by integrating metabolomics, machine learning, and *in vitro* functional assays, we characterized RAO by systemic immune imbalance and specific fatty acid disruptions, notably within the C22-series. Crucially, upon stratifying patients with RAO by dyslipidemia, C20:2*n*-6 emerged as a core dyslipidemic signature that drives Treg proliferation and specific IL-10 secretion. These findings highlight the critical crosstalk between fatty acid metabolism and immune pathways. We identified C20:2*n*-6 as a robust diagnostic biomarker and propose the C20:2n-6–Treg–IL-10 axis as a promising therapeutic target for dyslipidemia-associated RAO.

### Limitations of the study

Although this study provides a robust framework for identifying innovative biomarkers and understanding immunometabolic mechanisms, several limitations exist. First, although using cell experiments, we established a direct functional link; the clinical component of this study utilized a cross-sectional design, which precludes establishing causal relationships between systemic metabolic shifts and the onset of RAO. Second, our immunologic inferences were mainly drawn from PBMC-based readouts and therefore reflect systemic immune reprogramming, which may not fully capture the compartmentalized immune milieu within the retinal microvasculature and ischemic retina. Accordingly, tissue-level investigations are required to verify whether the C20:2n-6–Treg–IL-10 axis also operates locally at the disease site. Third, although our total cohort (*n* = 132) was substantially large, the RNA-seq component was limited to 37 subjects (19 patients with RAO and 18 controls). This small sample size for transcriptomics may have reduced the detection power for subtle gene-metabolite interactions within specific subgroups. Due to this limitation, we applied nominal *p* values rather than strict FDR correction. While this strategy increases the risk of false positives, the key identified mechanism of Treg immunometabolic dysfunction was subsequently validated by *in vitro* experiments, supporting the biological reliability of our findings despite statistical limitations. In the future, we should prioritize *in vivo* animal models to validate the therapeutic potential of the C20:2n-6–Treg–IL-10 axis and recruit larger, multi-center cohorts to substantiate the clinical generalizability of these findings.

## Resource availability

### Lead contact

Requests for further information and resources should be directed to and will be fulfilled by the lead contact, Xuan Xiao (xiaoxuan1111@whu.edu.cn).

### Materials availability

This study did not generate new unique reagents.

### Data and code availability

• Data: The raw bulk RNA-sequencing data reported in this study have been deposited in the National Genomics Data Center, China National Center for Bioinformation, under BioProject accession number BioProject: PRJCA058832. The raw targeted metabolomics data have been deposited in iProX, a ProteomeXchange consortium member repository, under accession number ProteomeXchange: PXD077366 (Project ID: iProX: IPX0016636000; Subproject ID: iProX: IPX0016636001). Processed transcriptomic data, integrative multiomics analysis files, and other supporting datasets have been deposited in Dryad and are publicly available at Dryad: https://doi.org/10.5061/dryad.98sf7m0wt.
• Code: The code used for transcriptomic, metabolomic, and integrative analyses has been deposited in Dryad and is publicly available at Dryad: https://doi.org/10.5061/dryad.98sf7m0wt.
• Additional information: Any additional information required to reanalyze the data reported in this article is available from the lead contact upon request.

## Acknowledgments

The authors would like to express their gratitude to all participants involved in the study. We also wish to acknowledge the Eye Institute of Renmin Hospital of Wuhan University for providing the experimental facilities and environment, and Renmin Hospital of Wuhan University for technical support. This work was supported by the 10.13039/501100012166National Key R&D Program of China (2023YFC2308404), the Health Commission of Hubei Province Scientific Research Project (HBJG-250010), and the 10.13039/501100003819Natural Science Foundation of Hubei Province (2022CFC066).

## Author contributions

J.F.: methodology, visualization, formal analysis, writing - original draft, and writing - review and editing; D.C.: data curation, formal analysis, writing - original draft, and writing - review and editing; C.W.: visualization and writing - review and editing; R.Z.: investigation; L.C.: validation; L.H.: software; T.C.: resources; Y.L.: project administration and writing - review and editing; X.X.: conceptualization, funding acquisition, and supervision.

## Declaration of interests

The authors declare no competing interests.

## STAR★Methods

### Key resources table

**Table:** 

REAGENT or RESOURCE	SOURCE	IDENTIFIER
**Biological Samples**		

Human peripheral blood samples	Renmin Hospital of Wuhan University	This paper

**Chemicals, Peptides, and Recombinant Proteins**		

C20:2*n*-6 (*cis*-11,14-eicosadienoic acid)	Sigma-Aldrich	Cat# E3127
Lymphocyte separation medium	MP Biomedicals	Cat# 50494×
CFSE	Elabscience	Cat# E-CK-A345
Human IL-10 ELISA Kit	Elabscience	Cat# E-EL-H6154
Human TGF-β1 ELISA Kit	Elabscience	Cat# E-EL-0162
RPMI Medium 1640 basic	Gibco	C11875500BT
Trizol reagent	Vazyme	R411-01
Fetal Bovine Serum (FBS)	Cegrogen	Cat# A0500-3011

**Experimental Models: Cell Lines**		

MT-2 (HTLV-1 transformed T cell)	Shanghai Yu Bo Biotech Co., Ltd	RRID:CVCL_1407

**Software and Algorithms**		

SPSS v27.0	IBM	RRID:SCR_002865
R v4.2.3	R Foundation	RRID:SCR_001905
FlowJo v10.8.1	FlowJo, LLC	RRID:SCR_008520
MetaboAnalyst	Xia Lab, McGill University	https://www.metaboanalyst.ca/RRID:SCR_015539
CIBERSORT	Alizadeh Lab, Stanford University	https://cibersort.stanford.edu/RRID:SCR_016955

**Deposited data**		

Raw bulk-RNA sequencing data	NGDC	BioProject: PRJCA058832
Raw targeted metabolomics data	iProX	ProteomeXchange: PXD077366; iProX: IPX0016636000; iProX: IPX0016636001
Targeted metabolomics data	Dryad	Dryad: https://doi.org/10.5061/dryad.98sf7m0wt
Processed data (differential expression lists, etc.)	Dryad	Dryad: https://doi.org/10.5061/dryad.98sf7m0wt
Original analysis code	Dryad	Dryad: https://doi.org/10.5061/dryad.98sf7m0wt

### Experimental model and study participant details

#### Human sample

Blood samples were collected from 132 participants, including 66 patients with RAO and 66 cataract controls, at Renmin Hospital of Wuhan University, China. Among them, 19 patients with RAO and 18 cataract controls were enrolled for bulk RNA-sequencing (RNA-seq). The study was approved by the Ethics Committee of Wuhan People’s Hospital (protocol code, WDRY2022-K278; approval date, November 30, 2022), adhering to the 1995 Declaration of Helsinki guidelines. All participants provided informed consent. Detailed demographic characteristics, including age and sex, are provided in Table 1. In this study, we analyzed the association of sex with the results. Information regarding race and ethnicity was not collected as the study population is homogeneous (Han Chinese).

#### Cell lines

The human T cell leukemia virus type 1 (HTLV-1) transformed regulatory T cell line (MT-2, ATCC CRL-1454) was purchased from Shanghai Yu Bo Biotech Co., Ltd (Shanghai, China) and authenticated by STR profiling. The cells were routinely tested for mycoplasma contamination and confirmed to be negative prior to experiments. The cell line is of male origin. MT-2 cells were cultured in RPMI-1640 medium (C11875500BT, Gibco) supplemented with 12% fetal bovine serum (Cat. No. A0500-3011, Cegrogen) at 37°C in a 5% CO2 atmosphere. The cell line was obtained from a commercial source and was not authenticated by the authors.

### Method details

#### Subject recruitment and classification

The patients with RAO were eligible for inclusion if they had a confirmed clinical and angiographic diagnosis within 72 h of symptom onset, a best-corrected visual acuity (BCVA) between counting fingers and 20/50, and available peripheral vein samples prior to thrombolysis. Exclusion criteria included glaucoma, retinal or optic disk neovascularization, prior RAO treatments, non-RAO vascular retinopathies, intraocular surgery within the last three months, stroke, myocardial infarction, hemi-RAO, macula-sparing RAO, or other severe ocular conditions. The RAO group met established diagnostic criteria, including acute vision loss, a positive relative afferent pupillary defect, retinal ischemic edema, and delayed arterial filling on angiography.^1^

Cataract control subjects underwent surgery within the same time frame. For both groups, additional exclusion criteria were a history of RAO, coronary artery disease, malignancy, severe renal impairment (estimated glomerular filtration rate <30 mL/min), liver disease, stroke, or pulmonary disease.

Participants were classified as having dyslipidemia if they met at least one of the following criteria based on fasting blood lipid measurements: total cholesterol (TC) ≥ 6.2 mmol/L, low-density lipoprotein cholesterol (LDL-C) ≥ 4.1 mmol/L, triglycerides (TG) ≥ 2.3 mmol/L, or high-density lipoprotein cholesterol (HDL-C) < 1.0 mmol/L.^25^

#### Metabolite extraction

Blood samples collected for metabolomic analysis were centrifuged at approximately 1000 × g for 20 min at 20°C–25°C. The serum was aliquoted and stored at −80°C until extraction. Samples were slowly thawed at 4°C, and a suitable amount of sample was added to 5 mL of dichloromethane-methanol solution (2:1 v/v) and mixed by vortexing. The samples were washed with 2 mL of distilled water, and the lower phase was collected. The solvent was dried under nitrogen. Then, 2 mL of n-hexane was added, followed by the internal standard, and the mixture was methylated for 30 min. Thereafter, 2 mL of distilled water was added, and the upper phase (2000 μL) was collected, dried under nitrogen, and reconstituted in n-hexane. The reconstituted solution was transferred to an injection vial for analysis. A 1 μL sample was injected into a gas chromatography-mass spectrometry (GC-MS) system with a 10:1 split ratio.

#### GC-MS conditions

Chromatography conditions: Separation was performed using a capillary column (Agilent 19091S–433UI:HP-5ms, 30 m × 250 μm × 0.25 μm) on an Agilent gas chromatograph. The temperature program was as follows: initial temperature, 80°C, ramp at 20°C/min to 180°C and held at this temperature for 8 min; further ramp at 5°C/min to 280°C and held at this temperature for 3 min. Helium was used as the carrier gas at a flow rate of 1.0 mL/min. A quality control (QC) sample was included at regular intervals within the sample batch to assess the stability and reproducibility of the system.

Mass spectrometry conditions: Mass spectrometry was performed using a 5977B MSD mass spectrometer (Agilent). The following conditions were set: injector temperature, 280°C; ion source temperature, 230°C; and transfer line temperature, 250°C. Electron ionization (EI) was performed at 70 eV, and both SCAN and SIM modes were used for detecting target analytes. Information for all target compounds is provided in Table S1.

Chromatographic peak areas and retention times were extracted using the MSD ChemStation software. Calibration curves were constructed to calculate the concentrations of MLCFAs in the samples.

#### Isolation of PBMCs and RNA-seq library construction

For RNA-seq, fresh blood samples from patients with RAO and controls were collected in EDTA anticoagulant tubes. PBMCs were isolated via density gradient centrifugation using Lymphocyte Separation Medium (Cat# 50494×, MP Biomedicals) according to the manufacturer’s protocol. Total RNA extracted from PBMC pellets using TRIzol reagent was used for the preparation of sequencing libraries. RNA quality was assessed for purity, concentration, and integrity.

Three micrograms of total RNA was used as input. mRNA was purified and fragmented. cDNA was synthesized and amplified to generate sequencing libraries. Following final library QC on the Agilent Bioanalyzer to confirm the expected size distributions, the libraries were pooled and sequenced on a DNBSEQ-T7 platform using 150 bp paired-end reads. Raw RNA-seq reads were processed, and gene-level read counts were generated using the featureCounts function in the Subread package (v1.5.3) with the following parameters: -t exon -g gene_id -s 0 -p.

#### *In vitro* functional assays

To evaluate the immunomodulatory effects of *cis*-11,14-eicosadienoic acid (C20:2*n*-6), cells were labeled with CFSE (Cat. No. E-CK-A345, Elabscience) following the manufacturer’s protocol. The labeled cells were seeded and treated with C20:2*n*-6 (Cat. No. E3127, Sigma-Aldrich) at final concentrations of 0, 10, 25, 50, 100, and 200 μM for 12 h. Subsequently, the medium was replaced with fresh fatty acid-free medium. Cell culture supernatants were collected 24 h after medium replacement for the quantification of IL-10 and TGF-β using ELISA kits (Human IL-10: E-EL-H6154; TGF-β1: E-EL-0162; Elabscience) according to standard procedures. Cell proliferation was assessed by measuring CFSE fluorescence intensity via flow cytometry (CytoFLEX LX, Beckman Coulter) at 48 h. Data analysis was performed using the FlowJo software (v10.8.1).

### Quantification and statistical analysis

#### Statistical analysis

Baseline clinical characteristics and *in vitro* functional assay data were analyzed using SPSS (v27.0; IBM, USA) and GraphPad Prism (v10.1.2; GraphPad Software, USA). For clinical data, continuous variables are expressed as mean ± SD (normally distributed, analyzed using the *t* test) or median (interquartile range) (non-normally distributed, analyzed using the Mann–Whitney U test). Categorical variables were compared via chi-square tests. For *in vitro* experiments, data are presented as the mean ± SD of independent biological replicates. Statistical significance between two independent groups was evaluated using an unpaired two-tailed Student’s *t* test, whereas comparisons involving multiple groups were assessed using a one-way analysis of variance (ANOVA) followed by Dunnett’s multiple comparisons test. A two-sided *p* < 0.05 was considered statistically significant.

#### Metabolomics data analysis

Fatty acid concentration matrices were analyzed using MetaboAnalyst for multivariate analyses, including sparse partial least squares discriminant analysis (sPLS-DA), orthogonal partial least squares-discriminant analysis (OPLS-DA), and correlation analysis. OPLS-DA identified principal components driving intergroup differences, with variable importance in projection (VIP) scores calculated for each fatty acid. Features with VIP >1.0 and significant concentration differences (*p* < 0.05) were classified as differential metabolites.^43^ Pathway enrichment analysis was conducted using the Relational Database of Metabolomic Pathways (RaMP-DB).

Four machine-learning models—support vector machines (SVM),^44^ random forest (RF),^45^ extreme gradient boosting (XGBoost),^46^ and light gradient boosting machine (LightGBM)^47^—were trained with hyperparameter tuning and quintuple cross-validation to evaluate the predictive performance of biomarkers. Receiver operating characteristic (ROC) curves were generated, with area under the ROC curve (AUC) > 0.65 indicating significant predictive potential.

#### Transcriptomics data analysis

RNA-seq data were normalized (quantile normalization) and processed using the limma package (R v4.2.3).^48^ Differential expression analysis revealed differentially expressed genes (|log2FC| ≥ 0.585, *p* < 0.05) via volcano plots. Principal component analysis (PCA) was used to visualize group differences. Gene ontology (GO) and Kyoto encyclopedia of genes and genomes (KEGG) pathway enrichment analyses were performed using clusterProfiler (adjusted *p* < 0.05).^49^ Immune cell abundance (22 cell types) was quantified from mRNA expression matrices using the CIBERSORT algorithm.^50^ Results were visualized as boxplots (ggplot2 R package).

#### Multiomics integration analysis

To investigate systemic interactions between metabolic dysregulation and immune responses in RAO, we integrated metabolomic and transcriptomic data. Curated immune signature gene sets (29 predefined signatures) were combined with PBMC RNA-seq data to quantify immune feature activity.^51^ For each immune signature, the activity score was calculated as the mean normalized expression of its constituent genes per sample. These scores were compiled into an immune activity matrix across the cohort. Spearman’s rank correlation was then applied to assess pairwise relationships between immune activity scores and serum fatty acid metabolite levels, aimed at identifying potential mechanistic links between lipid metabolism and immune pathways in RAO pathogenesis.

## Supplemental information

Document S1. Language_editing_certificate

Table S1. Information for all target fatty acid, related to Figure 1, 2, 3 and 4
