# Supplementary material for: Multiomics reveals fatty acid metabolism and immune remodeling in retinal artery occlusion
Source: iScience. 2026 Jun 17;29(7):116445. doi: 10.1016/j.isci.2026.116445 (PMC13293730; doi:10.1016/j.isci.2026.116445)
Supplement: Document S1. Language_editing_certificate [file mmc1.pdf]

iScience, Volume *29*

Supplemental information

Multiomics reveals fatty acid metabolism and immune remodeling in retinal artery occlusion

Jiaqing Feng, Duan Chen, Chuansen Wang, Runlang Zhu, Longfei Chen, Liang Hu, Ting Chen, Ying Li, and Xuan Xiao

Certificate of Elsevier Language Editing Services

The following article was edited by Elsevier Language Editing Services:

Fatty Acid Metabolism Reprograms Immune Microenvironment in Retinal Artery Occlusion: Multi-Omics Analysis Highlights Immunometabolic Crosstalk

Ordered by:

Duan Chen

Estimated Delivery date:

2026-02-13

Order reference:

ASLESTD1125163
