# Supplementary material for: Multiomics reveals fatty acid metabolism and immune remodeling in retinal artery occlusion
Source: iScience. 2026 Jun 17;29(7):116445. doi: 10.1016/j.isci.2026.116445 (PMC13293730; doi:10.1016/j.isci.2026.116445)
Supplement: Table S1. Information for all target fatty acid, related to Figure 1, 2, 3 and 4 [file mmc2.pdf]

Metabolite Name	Abbreviation	Retention Time(min)	KEGG ID	HMDB ID	CAS
Butyric acid	c4:0	0	C00246	HMDB0000039	107-92-6
Hexanoic acid	c6:0	5.953946769	C01585	HMDB0000535	142-62-1
Octanoic acid	c8:0	7.045985204	C06423	HMDB0000482	124-07-2
Decanoic acid	c10:0	8.088707908	C01571	HMDB0000511	334-48-5
Undecanoic acid	c11:0	8.621700765	C17715	HMDB0000947	112-37-8
Lauric acid	c12:0	9.187399575	C02679	HMDB0000638	143-07-7
Tridecanoic acid	c13:0	9.802322704	C17076	HMDB0000910	638-53-9
Myristic acid	c14:0	10.49857253	C06424	HMDB0000806	544-63-8
Myristoleic acid	c14:1n5	10.81401284	C08322	HMDB0002000	544-64-9
Pentadecanoic acid	c15:0	11.28391667	C16537	HMDB0000826	1002-84-2
10Z-Pentadecenoic acid	c15:1n5	0	-	HMDB0304816	84743-29-3
Palmitic acid	c16:0	12.19283265	C00249	HMDB0000220	57-10-3
Palmitoleic acid	c16:1n7	12.48905842	C08362	HMDB0003229	373-49-9
Heptadecanoic acid	c17:0	13.18074872	-	HMDB0002259	506-12-7
10Z-Heptadecenoic acid	c17:1n7	0	-	HMDB0060038	29743-97-3
Stearic acid	c18:0	14.30405213	C01530	HMDB0000827	57-11-4
Elaidic acid	c18:1tn9	0	C01712	HMDB0000573	112-79-8
Oleic acid	c18:1n9	14.60893129	C00712	HMDB0000207	112-80-1
Linolelaidic acid	c18:2ttn6	0	-	HMDB0006270	506-21-8
Linoleic acid	c18:2n6	15.20766386	C01595	HMDB0000673	60-33-3
γ-Linoleic acid	c18:3n6	15.54580833	C06426	HMDB0003073	506-26-3
α-Linolenic acid	c18:3n3	15.94876284	C06427	HMDB0001388	463-40-1
Arachidic acid	c20:0	16.75090374	C06425	HMDB0002212	506-30-9
11Z-Eicosenoic acid	c20:1n9	17.09639966	C16526	HMDB0002231	5561-99-9
11Z,14Z-Eicosadienoic acid	c20:2n6	17.77171241	C16525	HMDB0005060	5598-38-9
8Z,11Z,14Z-Eicosatrienoic acid	c20:3n6	18.16182602	C03242	HMDB0002925	1783-84-2
11Z,14Z,17Z-Eicosatrienoic acid	c20:3n3	18.6050716	C16522	HMDB0060039	17046-59-2
Arachidonic acid	c20:4n6	18.41055009	C00219	HMDB0001043	506-32-1
5Z,8Z,11Z,14Z,17Z-Eicosapentaenoic acid	c20:5n3	19.40293214	C06428	HMDB0001999	10417-94-4
Heneicosanoic acid	c21:0	18.06296046	-	HMDB0002345	2363-71-5
Behenic acid	c22:0	19.2619142	C08281	HMDB0000944	112-85-6
Erucic acid	c22:1n9	19.47461769	C08316	HMDB0002068	112-86-7

13Z,16Z-Docosadienoic acid	c22:2n6	20.4925977	C16533	HMDB0061714	17735-98-7
Adrenic acid	c22:4n6	21.34242662	C16527	HMDB0002226	28874-58-0
7Z,10Z,13Z,16Z,19Z-Docosapentaenoic acid	c22:5n3	22.3869631	C16513	HMDB0006528	24880-45-3
4Z,7Z,10Z,13Z,16Z-Docosapentaenoic acid	c22:5n6	21.57146956	-	HMDB0001976	25448-00-4
4Z,7Z,10Z,13Z,16Z,19Z-Docosahexaenoic acid	c22:6n3	0	C06429	HMDB0002183	6217-54-5
Tricosanoic acid	c23:0	20.78761335	-	HMDB0001160	2433-96-7
Lignoceric acid	c24:0	22.37374974	C08320	HMDB0002003	557-59-5
Nervonic acid	c24:1n9	22.65229609	C08323	HMDB0002368	506-37-6

Table S1: Related to Figure 1, 2, 3 and 4. Information for all target fatty acid.
